# Caffeic Acid Prevented LPS-Induced Injury of Primary Bovine Mammary Epithelial Cells through Inhibiting NF-*κ*B and MAPK Activation

**DOI:** 10.1155/2019/1897820

**Published:** 2019-04-30

**Authors:** Mingjiang Liu, Guoqing Fang, Shaojie Yin, Xin Zhao, Chi Zhang, Jingui Li, Zongping Liu

**Affiliations:** ^1^College of Veterinary Medicine, Yangzhou University, Yangzhou 225009, China; ^2^Jiangsu Co-innovation Center for Prevention and Control of Important Animal Infectious Diseases and Zoonoses, Yangzhou 225009, China

## Abstract

In our previous study, lipopolysaccharide (LPS) significantly reduced the cell viability of primary bovine mammary epithelial cells (bMEC) leading to cell apoptosis, which were prevented by caffeic acid (CA) through inhibiting NF-*κ*B activation and reducing proinflammatory cytokine expression. While the underlying mechanism remains unclear, here, we determined that LPS induced the extensive microstructural damage of bMEC, especially the mitochondria and endoplasmic reticulum. Then, the obvious reduction of mitochondrial membrane potential and expression changes of apoptosis-associated proteins (Bcl-2, Bax, and casepase-3) indicated that apoptosis signaling through the mitochondria should be responsible for the cell viability decrease. Next, the high-throughput cDNA sequencing (RNA-Seq) and Kyoto Encyclopedia of Genes and Genomes (KEGG) pathway enrichment analysis were employed to verify that the MAPK and JAK-STAT signaling pathways also were the principal targets of LPS. Following, the critical proteins (ERK, JNK, p38, and c-jun) of the MAPK signaling pathways were activated, and the release of proinflammatory cytokines (TNF-*α*, IL-1*β*, IL-6, and IL-8) regulated by NF-*κ*B and MAPKs was significantly increased, which can promote a cascade of inflammation that induces cell injury and apoptosis. Meanwhile, CA significantly inhibited the activation of MAPKs and the release of proinflammatory cytokines in a dose-dependent manner, which were similar to its effects on the NF-*κ*B activation that we previously published. So we concluded that CA regulates the proteins located in the upstream of multiple cell signal pathways which can reduce the LPS-induced activation of NF-*κ*B and MAPKs, thus weakening the inflammatory response and maintaining cell structure and function, which accordingly inhibit apoptosis.

## 1. Introduction

Inflammation is a natural biological response of the body to stimuli such as tissue injury, pathogen invasion, and irritants. Clinical mastitis caused by *Escherichia coli* (*E. coli*) accounts for significant production losses and animal welfare concerns on dairy farms worldwide and can lead to the death of the animal [1–3]. This kind of excessive inflammation following stimulus participates in the pathological courses of lesion of mammary gland tissue and reduces the production performance of cows [2, 4, 5]. Therefore, the active control of the excessive inflammation is crucial for the treatment of bovine mastitis caused by *E. coli*.

Bovine mammary epithelial cells (bMEC) are the dominant cell type in the healthy, uninfected milk parenchyma, which are the most likely first cells to be confronted with a pathogen in the milk parenchyma [6]. The principal function of bMEC is milk formation during lactation; however, its immune regulation has become a hotspot for research in recent years [6–8]. Numerous studies have now shown that bMEC are able to sense bacteria or bacterial products and to make a strong response [6, 7, 9]. Another study obtained that bMEC could contribute to the onset of an early response of the mammary gland after infection by *E. coli* and play an important role in promoting neutrophil recruitment and enhance tissue inflammation during *E. coli* mastitis [10]. Our previous study confirmed that lipopolysaccharide (LPS) induced a rapid and strong inflammatory response in bMEC and upregulated several sets of genes involved in the innate immune response. The cell viability of bMEC was significantly inhibited as a result of cell apoptosis induced by LPS [11], but the exact molecular mechanisms of LPS-induced inflammatory injury of bMEC are still unclear.

It is a matter of prime importance to eradicate bacteria earlier and control the inflammation in mastitis, which would contribute to restore tissue homeostasis and prevent the occurrence of chronic inflammation [12]. Antibiotics are still an effective method for the treatment of bovine mastitis, but the use of them is restricted due to the growing problems of drug resistance and food safety [13, 14]. Additionally, antibiotics promote the substantial release of bacterial compounds which consequently enhance inflammation, and this highly limits the effects of antibiotics on reducing inflammation [15–17], so safe and effective treatments of bovine mastitis are of increasing interest in veterinary research. Caffeic acid (3,4-dihydroxycinnamic acid (CA)) is the major dietary hydroxycinnamic acid, which is a phenolic compound widely found in nature and possesses a number of biological activities such as antibacterial, antioxidant, anti-inflammatory, and anticancer growths [18–20]. Despite many studies reporting the anti-inflammatory properties of CA, the positive impact of CA on LPS-induced inflammation injury of bMEC was preliminarily confirmed in a previous study [11], but the exact molecular mechanisms remain unclear.

## 2. Materials and Methods

### 2.1. bMEC Isolation, Cell Culture, and Treatment

bMEC were isolated from lactating cows whose four quarters were free from pathogens, and somatic cell counts were under 150,000 cells/mL of milk for all the 4 quarters. The method for isolating and culturing bMEC was according to a previous study [21] with little modifications. Immediately after slaughter, the udder was removed and the secretory tissue was taken from the right back quarter. Approximately 10 g sections were minced and incubated under agitation in 30 mL of culture media with collagenase from Clostridium histolyticum (2 mg/mL; Sigma-Aldrich, St. Louis, MO, USA). After 2 hours, the mixture was filtered through a 200 *μ*m copper wire mesh to collect dispersed cells. Cells were incubated at 37°C in 5% CO_2_. The complete culture medium consisting of DMEM/F12 media supplemented with 10% fetal bovine serum (Gibco, Grand Island, NY), 100 U/mL antibiotic (penicillin and streptomycin, Sigma-Aldrich), 5 *μ*g/mL insulin, 1 *μ*g/mL hydrocortisone, 5 *μ*g/mL transferrin, and 1 *μ*g/mL progesterone (Sigma-Aldrich) was used at passage 3 or 4 for further research.

When reaching 90% confluence, cells were washed twice with phosphate-buffered saline (PBS) and then stimulated with LPS (50 *μ*g/mL) for indicated times (0.5, 1, 1.5, and 12 h) following incubation with CA (0, 10, 25, and 50 *μ*g/mL) for 3 h. Next, cells were washed three times with PBS and used for the following experiments: the selections of LPS and CA doses were according to a previous study [11]. CA (National Institutes for Food and Drug Control, >98% purity, Beijing, China) and LPS (*E. coli* serotype O55:B5, Sigma-Aldrich, St. Louis, MO, USA) were diluted in DMEM/F12 medium to a final concentration of 1 mg/mL before being added to culture media to achieve the final concentration required in respective assays.

### 2.2. Scanning Electron Microscopy (SEM) and Transmission Electron Microscopy (TEM)

For SEM analysis, cells were planted on cover glasses (25 × 25 mm) which were placed in 6-well multiplies. After being pretreated with CA for 3 h, cells were stimulated with LPS for 12 h, cover slips removed, and cells fixed with 3% glutaraldehyde (room temperature) for 24 h. Fixed cells were rinsed with PBS and then dehydrated in EtOH (70%>80%>90%>95%>100%) and dried in a critical point dryer (Hitachi SCP-II). After coating with gold using an IB-5 ion coater (Eiko), cells were observed under SEM (S-570, HITACHI, Japan).

For TEM analysis, cells were fixed with 3% glutaraldehyde at 20°C for 48 h. Following fixing, cells were washed and dehydrated as before, and cells were embedded in Epon-Araldite mix solution and blocked at 60°C in a vacuum drying oven (Yamoto, DPF-31) for 36 h. First, semithin slides were made using an ultramicrotome (LKB-2088) and stained with 1% toluidine blue (1% borax) on a 60°C hot plate for 2 min. Then, ultrathin slices were made and stained with uranyl acetate and lead citrate. The cell microstructures were observed under TEM (JEM-1230, JEOL, Japan).

### 2.3. Cell Apoptosis and Mitochondrial Membrane Potential Evaluation

Cells were placed in 6-well multiplies, after being pretreated with CA for 3 h; cells were stimulated with LPS for 12 h. To analyze apoptosis, the cells were trypsinized (Gibco, Grand Island, NY), washed twice with PBS, and then stained with annexin V/propidium iodide (Invitrogen Inc., Carlsbad, CA), and flow cytometric analysis was performed according to the manufacturer's instructions (Becton Dickinson, San Jose, California).

The mitochondrial membrane potential (ΔΨm) was measured using the mitochondrial potential sensor 5,5′,6,6′-tetra-chloro-1,1′,3,3′-tetra-ethyl-benz-imidazolyl-carbocyanine iodide (JC-1, Jiancheng, Nanjing, China). The cells were collected and incubated with 4 *μ*M JC-1 for 20 minutes at 37°C in the dark. Extracellular JC-1 was removed by washing cells twice in PBS; then, the amount of JC-1 retained by 20,000 cells per sample was analyzed on a flow cytometer using 488 nm excitation with 530 nm and 585 nm bandpass emission filters (Becton Dickinson, San Jose, California).

### 2.4. RNA Extraction, RNA Sequencing, and De Novo Transcriptome Assembly

After being pretreated with CA for 3 h, cells were stimulated with LPS for 12 h. Then, the total RNA was extracted using the TRIzol Reagent (Invitrogen Inc.) and treated with DNase; then, the quality and quantity of the purified RNA were determined using a NanoDrop 2000c spectrophotometer (Thermo Fisher, Holliston, MA) [22]. Its integrity was assessed by measuring the RNA integrity number (RIN) using a 2100 Bioanalyzer (Agilent, Santa Clara, CA), and the Illumina high-throughput cDNA sequencing (RNA-seq) experiment and the transcriptome assembly analysis were conducted at BGI Tech (Shenzhen, China).

### 2.5. Western Blot Analysis

Cells were stimulated with LPS for 0.5, 1, and 1.5 h after being pretreated with CA for 3 h; then, the proteins were extracted using a total protein extraction kit (BioChain Institute Inc., Hayward, CA) and quantified using a BCA protein assay kit (Pierce Biotechnology Inc., IL). Western blot analysis was performed using equal quantities (10-20 *μ*g) of cell extracts, diluted in sample buffer, and separated using 4-12% gradient SDS-PAGE gels. Then, proteins were transferred to PVDF membranes (Millipore, Biotechnology Inc.) and hybridized with specific antibodies. The following primary antibodies were from Cell Signaling Technology, Danvers, MA: c-Jun NH2-terminal kinase 1/2 activation (JNK, #9258), phospho-JNK (#4668), p38 (#8690), phospho-p38 (#4511), extracellular signal-regulated kinase (ERK, #4695), phospho-ERK (#4370), c-Jun (#9165), phospho-c-Jun (#3270), and *β*-actin (#4970). The following primary antibodies were from Abcam, Cambridge, UK: Bcl-2 (ab183656), Bax (ab32503), and casepase-3 (ab90437). The stripes were detected using an Odyssey Infrared Imaging System (LI-COR Biosciences, Lincoln, NE). In all cases, *β*-actin was used as a loading control. Densitometric values of immunoblot signals were quantified using ImageJ.

### 2.6. ELISA Analysis

Cells were placed in 6-well multiplies, after being pretreated with CA for 3 h; cells were stimulated with LPS for 12 h. Then, the cell culture supernate was collected and stored in -20°C. The expression of TNF-*α*, IL-1*β*, IL-6, and IL-8 was analyzed using CUSABIO (Huamei, Wuhan, China) ELISA kit, according to the manufacturer's instructions. Absorbance values were measured at 450 nm, and the inflammation factor concentrations were calculated with reference to their standard curves. The detection range of TNF-*α* is 0.1 ng/mL-20 ng/mL, the detection range of IL-1*β* is 62.5 pg/mL-4000 pg/mL, the detection range of IL-6 is 5 pg/mL-1000 pg/mL, and the detection range of IL-8 is 50 pg/mL-2000 pg/mL.

### 2.7. Statistical Analysis

Unless others are indicated, all data were obtained from at least three independent experiments performed in triplicate and all data were analyzed using SPSS 20.0 statistical software. Data were performed using one-way ANOVA followed by Duncan's test for multiple comparisons and presented as the mean and standard error of the mean (Sem). For all analyses, *P* value of 0.05 or 0.01 was considered statistically significant.

## 3. Results

### 3.1. CA Weakened LPS-Induced Structural Damage of bMEC

**Figure 1:**
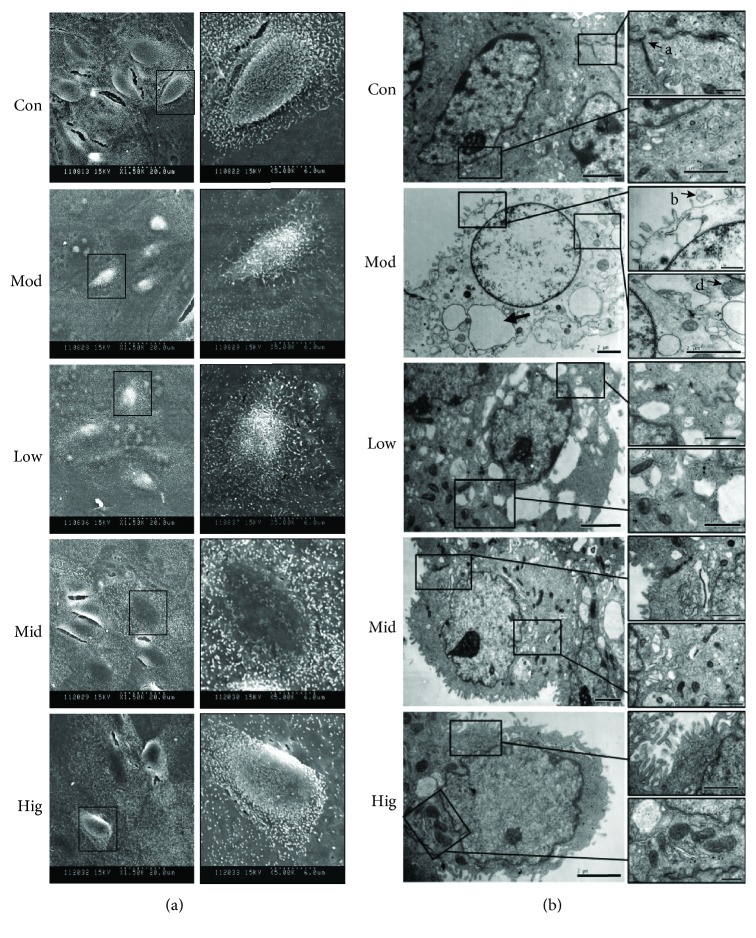
CA weakened LPS-induced structural damage of bMEC. The cell microstructure of bMEC after incubation with LPS or different concentrations of CA. Cells were pretreated with indicated concentrations (10, 25, and 50 *μ*g/mL) of CA or serum-free media for 3 h before stimulation with LPS (50 *μ*g/mL) for 12 h; then, the microstructure was observed by SEM and TEM. (a) Compared with the Con group, the phenomenon of cell crimple and cell microvilli disorders was obviously in the Mod group; however, these changes could be improved by CA in a dose-dependent manner. The images display the view in the left panel at 1500 times actual size and the view in the right panel at 5000 times actual size. (b) The swelling or rupture of microvilli (B), extreme expansion of endoplasmic reticulum (C), and swelling and hazy of mitochondrion (D) exist in the Mod group cells, and the electron density of cytoplasmic matrix structure was more lower than that of the other groups. CA significantly improved the above features, especially the medium and high dosage. The cell tight junction in the Hig group was clearly observed as in the Con group (A), which almost disappeared in the Mod group. Con: cells without any processing; Mod: cells treated with LPS (50 *μ*g/mL) only; Low: cells treated with LPS (50 *μ*g/mL) after incubation with 10 *μ*g/mL CA; Mid: cells treated with LPS (50 *μ*g/mL) after incubation with 25 *μ*g/mL CA; Hig: cells treated with LPS (50 *μ*g/mL) after incubation with 50 *μ*g/mL CA.

A previous study had determined that LPS significantly inhibited cell viability in a time- and dose-dependent manner [11]. In this study, after treatment with LPS (50 *μ*g/mL) for 12 h, the cell shape and structure were damaged including fuzzy cell boundary, cellular atrophy, disorderly or missing microvilli, and different degrees of cell collapse as observed by SEM (Figure 1(a)), and swelling or rupture of microvilli, nuclear cavitation, extreme expansion of endoplasmic reticulum, hazy mitochondrial structures, and loose cytoplasmic matrix structure were studied by means of TEM (Figure 1(b)), while CA effectively prevented the damage of cell morphology and microstructure induced by LPS in a dose-dependent manner and delivered a better impact at higher doses (Figure 1), indicating that the effect of CA on the maintenance of cell viability depends on its protective effect on the cell structure.

### 3.2. CA Inhibited the Decreasing of bMEC Mitochondrial Membrane Potential (ΔΨm) and Reduced the Cell Apoptosis Induced by LPS

**Figure 2:**
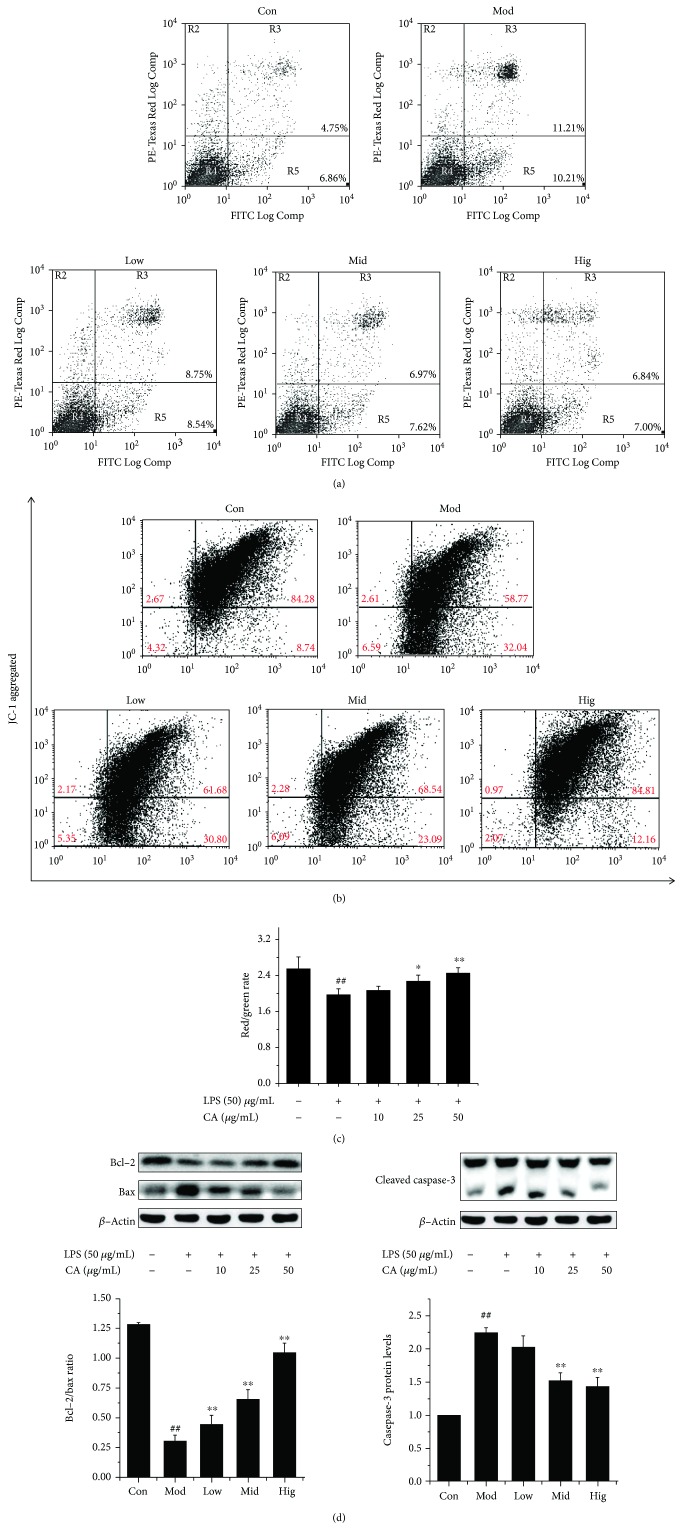
CA decreased cell apoptosis through inhibiting the mitochondria apoptosis pathway caused by LPS. The changes in apoptosis, mitochondrial membrane potential, and apoptosis-related protein levels of bMEC after treatment with LPS or CA. Cells were pretreated with indicated concentrations (10, 25, and 50 *μ*g/mL) of CA or serum-free media for 3 h before stimulation with LPS (50 *μ*g/mL) for 12 h. (a) The cell apoptosis was measured by flow cytometry following annexin V/propidium iodide (PI) staining. Cells that were positive for annexin V, but not PI, which are present in the lower right quadrant, were early apoptotic cells. Cells that were both annexin V and PI positive, which are present in the upper right quadrant, were late apoptotic cells. (b) Bivariate JC-1 analysis of mitochondrial membrane potential (ΔΨm) in cells by flow cytometry. Fluorescence of cells stained with JC-1 for the indicating treatments. In nondamaged cells, JC-1 forms red-emitting aggregates in the mitochondrial matrix, which are present in the upper right quadrant (high ΔΨm). A loss of red fluorescence and an increase in cytoplasmic green-emitting monomers signal the disruption of ΔΨm, which are present in the lower right quadrant (low ΔΨm). (c) The rate of red to green fluorescence intensity in different groups. The values represent the magnitude of ΔΨm; the data represent the means of 3 independent experiments. ^##^*P* < 0.01 vs. the control group, ^∗^*P* < 0.05 vs. the group treated with LPS only, and ^∗∗^*P* < 0.01 vs. the group treated with LPS only. (d) The total cellular proteins were collected; then, the protein levels of Bcl-2, Bax, and casepase-3 were detected by western blotting. Upper panel: western blot was performed to related protein levels; one out of three independent experiments is shown. Lower panel: quantification of related proteins normalized to actin levels. Data are shown as the means ± Sem (*n* = 3). ^##^*P* < 0.01 vs. the control group; ^∗∗^*P* < 0.01 vs. the group treated with LPS only.

After stimulation with LPS (50 *μ*g/mL) for 12 h, both early and late apoptosis were obviously increased (Figure 2(a)), which was consistent with a previous experiment result [11]. Besides, cell ΔΨm was significantly reduced (Figures 2(b) and 2(c)), as cells underwent a progressive loss of red fluorescence, while normal cells with well-polarized, red-emitting mitochondria were localized in the upper region of the plot, indicating a functional lesion in the mitochondria which was closely related to cell apoptosis. As shown in Figure 2(d), the rate of Bcl-2/bax significantly decreased (*P* < 0.01) and cleaved caspase-3 content was obviously elevated (*P* < 0.01), indicating the start of cell apoptotic process, which agreed with the result in Figure 2(a).

CA efficiently decreased the repression of cell ΔΨm and inhibited apoptosis induced by LPS in a dose-dependent manner, with high doses almost eliminating the negative influence of LPS (Figures 2(a)–2(c)) in line with its effects on the cellular structure (Figures 1(a) and 1(b)) and cell viability.

### 3.3. LPS Mainly Activated the NF-*κ*B, MAPK, and JAK-STAT Signaling Pathways of bMEC

**Figure 3:**
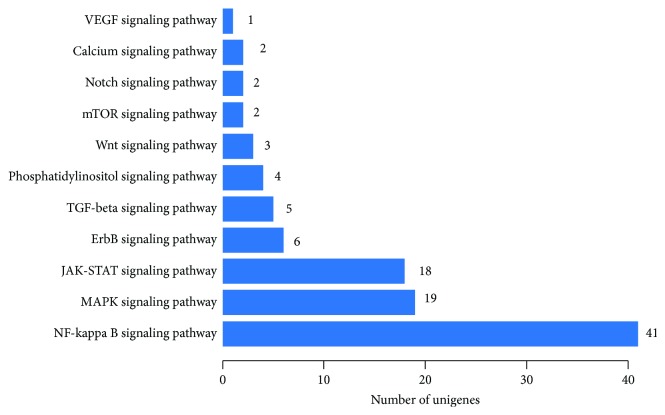
LPS mainly activated the NF-*κ*B, MAPK, and JAK-ATAT signaling pathways of bMEC. The number of differentially expressed genes relates to cell signaling transduction in LPS-stimulated bMEC. The total RNA was prepared after stimulation with LPS or without any processing for 12 h; then, the RNA-seq experiment and transcriptome assembly analysis were performed by BGI Tech. With significant pathway enrichment, we can ascertain the main biochemical pathways and signal transduction pathways which differentially expressed genes take part in. All the genes were used for the KEGG ontology (KO) enrichment analyses. For KO enrichment analysis, a *Q* value ≤ 0.05 was used as the threshold to determine significant enrichment of the gene sets.

To gain insight into the molecular mechanisms of bMEC damage in the context of LPS-mediated inflammation, we chose the RNA-Seq and Kyoto Encyclopedia of Genes and Genomes (KEGG) pathway enrichment approach to identify the main LPS-activated signal transduction pathways in bMEC. As shown in Figure 3, the identified significantly differential genes were mainly enriched in NF-*κ*B, MAPK, and Janus kinase/signal transducer and activator of transcription (JAK/STAT) signaling pathways, indicating that the NF-*κ*B signaling pathway is the principal target.

### 3.4. CA Inhibited the Activation of the MAPK Signaling Pathways in LPS-Stimulated bMEC

**Figure 4:**
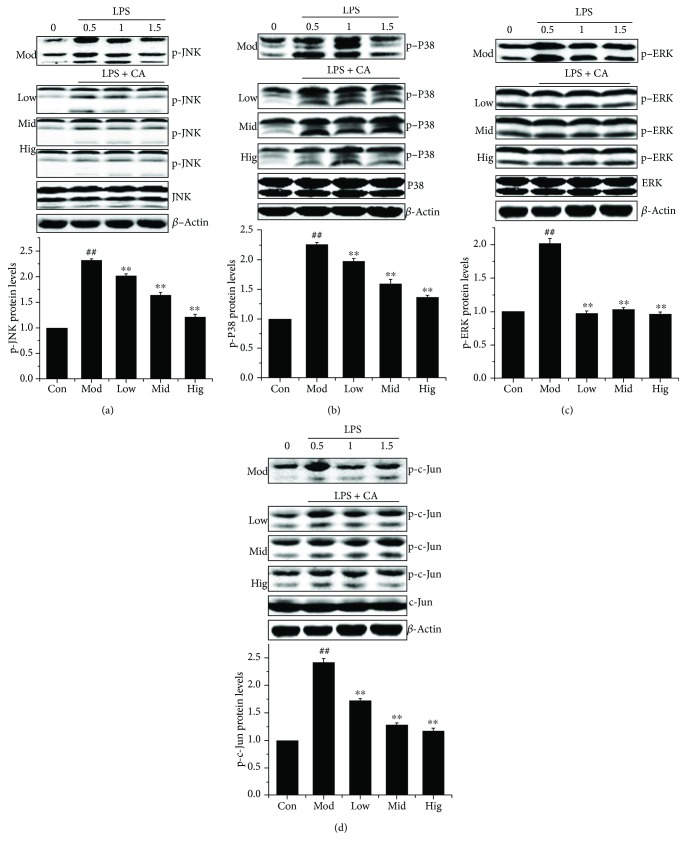
CA blocked the LPS-activated MAPK activation in bMEC. The effect of CA on the activation of critical proteins in the MAPK signaling pathways. Cells stimulated with LPS (50 *μ*g/mL) for 1.5, 1, and 0.5 h after incubation with indicated concentrations (10, 25, and 50 *μ*g/mL) of CA or serum-free media for 3 h; then, the total proteins were prepared and subjected to western blotting. Upper panel: western blot was performed to related protein levels; one out of three independent experiments is shown. Lower panel: quantification of related proteins normalized to actin levels (0.5 h). Data are shown as the means ± Sem (*n* = 3). ^##^*P* < 0.01 vs. the control group; ^∗∗^*P* < 0.01 vs. the group treated with LPS only.

We investigated the critical proteins of the MAPK signaling pathways in bMEC which were indeed activated by LPS. Western blot showed that CA pretreatment significantly inhibited the LPS-activated phosphorylation levels of JNK, p38, and c-Jun in a dose-dependent manner (Figures 4(a), 4(b), and 4(d)) and completely blocked the LPS-activated phosphorylation of ERK1/2 at various concentrations (Figure 4(c)). These findings suggested that CA inhibited the signaling cascades activated by LPS leading to MAPK activation.

### 3.5. CA Reduced the Release of Proinflammatory Cytokines in LPS-Stimulated bMEC

**Figure 5:**
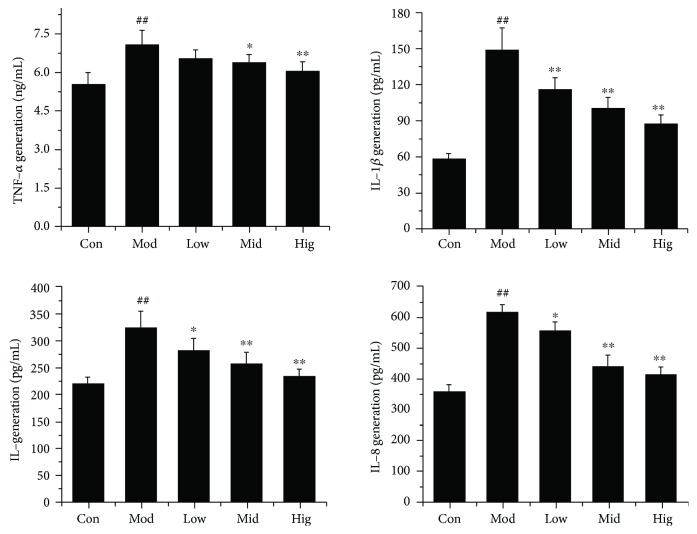
CA decreased LPS-induced proinflammatory cytokine release in bMEC. The effect of CA on the release of TNF-*α*, IL-1*β*, IL-6, and IL-8 in LPS-stimulated bMEC. Cells were challenged with LPS (50 *μ*g/mL) for 12 h after incubation with indicated concentrations (10, 25, and 50 *μ*g/mL) of CA or serum-free media for 3 h; subsequently, the cell culture supernate was prepared. The protein levels of TNF-*α*, IL-1*β*, IL-6, and IL-8 were quantified using ELISA kits; the data are the means ± Sem (*n* = 4). ^##^*P* < 0.01 vs. the control group, ^∗^*P* < 0.05 vs. the group treated with LPS only, and ^∗∗^*P* < 0.01 vs. the group treated with LPS only.

To further confirm the inhibitory effect of CA on LPS-induced inflammation in bMEC, the protein expression levels of IL-8, IL-1*β*, IL-6, and TNF-*α* were quantified. As expected, ELISA analysis indicated that LPS significantly (*P* < 0.01) increased the release of these proinflammatory cytokines, and the inhibiting effect of CA was dose-dependent (Figure 5).

## 4. Discussion

The host immune response and toxins produced by mastitis-causing bacteria are the causal mechanisms for the deleterious effects on mammary tissue. LPS, a major constituent of the outer membrane of gram-negative bacteria, is regarded as one of the most potent initiators of inflammation [16]. We had determined that LPS (50 *μ*g/mL) induced a significant reduction of bMEC viability in an early study [11]. Now, we proved that the profound morphological and microstructure changes (Figures 1(a) and 1(b)) induced by LPS were responsible for the cell viability reduction, rather than the cell growth inhibition or cell cycle arrest (data not shown). Presumably, cells experience severe functional impairments or even more. Besides, the decrease or even disappearance of intercellular tight junction (Figure 1(b)) may contribute to mammary epithelial cell desquamation and inflammatory cell infiltration. So there is no doubt that LPS plays an important role in mammary gland tissue damage in *E. coli* strains causing acute coliform mastitis, released in large quantities when the bacteria are killed by phagocytes and during exponential bacterial growth.

Mitochondria are the cellular hubs for metabolism, whose structure and function intact are crucial for cell health [23]. As shown in Figure 1(b), the mitochondrion structure was damaged after the stimulation with LPS; then, an obvious decrease of cell ΔΨm was observed (Figure 2(b)), which is used to assess mitochondrial function and cellular energy metabolism status and also an important hallmark of cell apoptosis [24–26]. LPS disrupted ΔΨm (more than 20% obvious reduction in a red/green fluorescence rate, Figure 2(c)), suggesting an important decrease in their respiratory chain activity or mitochondrial uncoupling, which induces outer membrane permeabilization leading to the release of proapoptotic factors into the cytoplasm and resulting in the apoptotic death of blasts [27]. So the analysis of cell apoptosis (Figure 2(a)) was intelligible which indicated that cells were dying, and this result was consistent with an early study [11]. The relative expression ratio of Bcl-2 and bax determines whether apoptosis happens in cells or not [28]; besides, caspase-3 is the most used target to detect apoptosis [29]. In Figure 2(d), the rate of Bcl-2/bax significantly decreased and cleaved caspase-3 content was obviously elevated, indicating the start of the cell apoptotic process, which agreed with the above results. The date indicated that the mitochondria apoptosis pathway plays a crucial role in LPS-induced bMEC injury.

CA is abundant in nature and has various biological activities [30]. In our study, we demonstrate that CA has remarkable protective effects on LPS-induced structural damage (Figure 1) and cell viability decrease in bMEC. CA administered at 50 *μ*g/mL almost entirely eliminated the damaging effects of LPS. To investigate the underlying mechanisms of CA protective action on bMEC, cDNA sequencing was used to reveal a significant enrichment of LPS potential targets in the NF-*κ*B, MAPK, and JAK-STAT signaling pathways (Figure 3). As it is well known, the NF-*κ*B and MAPK signaling pathways play an essential role in the innate immune response driving transcriptional activation of genes encoding proinflammatory cytokines, which act to mediate antigen-specific adaptive immune responses [31, 32], and we had proved that CA significantly inhibited the NF-*κ*B activation of bMEC induced by LPS [11]. So we investigated the effects of CA on LPS inducing the changes of the MAPK signaling pathways. As shown in Figure 4, after stimulation with LPS, the phosphorylation levels of JNK, p38, and ERK were all significantly increased (Figures 4(a)–4(c)), which were well-characterized subfamily members of the MAPK family. As MAPKs play an important role in the regulation of AP-1 activation [33, 34], the activity of AP-1 was detected, which is a heterodimeric transcription factor and normally functions as positive factors in regulating inflammation [35, 36]. AP-1 is composed of c-Fos, c-Jun, ATF, and JDP families, and c-Jun in combination with c-Fos forms the AP-1 early response transcription factor [34]. So the significant increase of p-c-Jun (Figure 4(d)) represented the enhancement of AP-1 activation in a certain degree. Meanwhile, these changes were significantly reversed by CA in a dose-dependent manner, indicating that CA can inhibit the LPS-induced activation of the MAPK signaling pathways. In consideration of the consistent effects of CA on these protein expression levels of the NF-*κ*B and MAPK signaling pathways, we presumed CA interacted with the upstream components of the LPS-activated signal-transmitting pathways.

Next, the release of various proinflammatory cytokines regulated by NF-*κ*B and AP-1 was also analyzed; as expected, CA significantly inhibited LPS-induced protein release of TNF-*α*, IL-1*β*, IL-6, or IL-8 in a dose-dependent way (Figure 5). Interestingly, the suppressive effects of CA on IL-1*β*, IL-6, or IL-8 production were greater than its effect on TNF-*α*. As we know, TNF exert their biological effects through the interaction with transmembrane receptors of the tumor necrosis factor receptor (TNFR) superfamily. On the one hand, recruitment of death domain- (DD-) containing adaptors such as Fas-associated death domain (FADD) and TNFR-associated DD (TRADD) can lead to the activation of a signal transduction pathway that induces apoptosis. On the other hand, the recruitment of tumor necrosis factor receptor-associated factor (TRAF) family proteins can lead to the activation of NF-*κ*B or AP-1 thereby promoting cell survival, differentiation, or death as well as immune and inflammatory responses [37, 38]. So TNF-*α* plays a central role in initiating and regulating the cytokine cascade during an inflammatory response [39, 40], and we concluded CA had an excellent prevention effect on the production of primary cytokines induced by LPS, thereby interfered with the inflammation cascade.

Depending on the cell type, TNF has been reported to trigger apoptosis through either a mitochondrial-dependent pathway or a mitochondrial-independent pathway [41, 42]. A TNF-initiated death signal is closely related with reactive oxygen species (ROS), which contribute to apoptosis by inducing mitochondrial permeability transition, uncoupling of oxidative phosphorylation, and depletion of ATP [23, 43]. Studies showed that ROS acts on many proteins needed to regulate cellular homeostasis, including those mediating cell proliferation, survival, death, differentiation, DNA repair, and metabolism [44, 45]. And there is now a rising interest in understanding the emerging function of TNF as a regulator of the generation of ROS and reactive nitrogen species (RNS), so this link between TNF and ROS adds another layer of complexity to the TNF signaling network [46]. In this study, we proved that the mitochondria pathway participated in LPS-induced bMEC apoptosis; besides, tests have shown that the mitochondria produced excessive amounts of ROS (data not shown). Clearly, superfluous ROS are also prone to causing oxidative damage and oxygen deprivation stress. So the oxidative stress may play an important role in LPS-induced bMEC injury, and the effects of CA on ROS signaling under physiological and pathophysiological conditions still need to be studied further.

The JAK-STAT signaling pathway is another relevant inflammatory pathway activated in response to cytokines [47, 48] and was activated in LPS-stimulated bMEC (Figure 3). There are seven STAT family members in mammals, which play essential roles in the development of the mammary gland with each having distinct functions in differentiation and milk production, programmed cell death, and immune cell-mediated tissue remodeling [49–51]. Meanwhile, in other tissues, each STAT protein has specific functions as a signal transduction molecule influencing cell growth, differentiation, development, and apoptosis and is involved in multiple steps in innate and adaptive immune responses [47, 52]. Studies indicated a general upregulation of genes associated with immune response mechanisms and downregulation of genes related to fat metabolism in the tissue of experimentally *E. coli*-induced mastitis quarters [53]. Such research findings were supported by the in vitro test looking at LPS-induced genic changes in bMEC (data not shown). The roles of the JAK-STAT signaling that participated in LPS-induced structural, functional, and apoptosis impairment of bMEC still need further research.

## 5. Conclusion

In summary, this study confirmed that CA effectively prevented LPS-induced bMEC injury and apoptosis in vitro through attenuating the inflammatory cascade reaction. The target proteins of CA may be located in the upstream of multiple cell signal pathways as the consistent effects on key proteins in the NF-*κ*B and MAPK pathways. In addition, the endoplasmic reticulum and mitochondria entail specific stress responses triggered by LPS played an important role in cellular functional and structural perturbations, which were closely related to inflammation and oxidative stress, but the cell signaling network remains largely unknown in bMEC. So the protection mechanisms of CA need to be studied further. What is more, the therapeutical effects of CA on LPS-induced bMEC injury need to be researched, which would provide support for CA as a promising candidate for the effective treatment of bovine mastitis-causing *E. coli*.

## Data Availability

The data used to support the findings of this study are available from the corresponding author upon request.
